# Bacterial Cellulose Membranes as Carriers for Nisin: Incorporation, Antimicrobial Activity, Cytotoxicity and Morphology

**DOI:** 10.3390/polym14173497

**Published:** 2022-08-26

**Authors:** Gabriela Ribeiro dos Santos, Victória Soares Soeiro, Carolina Fernanda Talarico, Janaína Artem Ataide, André Moreni Lopes, Priscila Gava Mazzola, Thais Jardim Oliveira, José Martins Oliveira Junior, Denise Grotto, Angela F. Jozala

**Affiliations:** 1 LAMINFE—Laboratory of Industrial Microbiology and Fermentation Process, University of Sorocaba, Sorocaba 18023-000, SP, Brazil; 2 Faculty of Pharmaceutical Science, University of Campinas (Unicamp), Campinas 13083-871, SP, Brazil; 3 LAFINAU—Laboratory of Nuclear Physics, University of Sorocaba, Sorocaba 18023-000, SP, Brazil; 4 LAPETOX—Laboratory of Toxicological Research, University of Sorocaba, Sorocaba 18023-000, SP, Brazil

**Keywords:** antimicrobial activity, bacterial cellulose, cytotoxicity, nisin, stability

## Abstract

Based on the previous study, in which nisin and bacterial cellulose were utilized, this new experiment loads nisin into bacterial cellulose (N–BC) and evaluates the morphological characteristics, cytotoxicity, antimicrobial activity and stability of the developed system. The load efficiency of nisin in BC was evaluated by an agar diffusion assay, utilizing *Lactobacillus sakei*, and total proteins. After having found the ideal time and concentration for the loading process, the system stability was evaluated for 100 days at 4, 25 and 37 °C against *Staphylococcus aureus* and *L. sakei*. Thus, in this study, there is a system that proves to be efficient, once BC has enhanced the antimicrobial activity of nisin, acting as a selective barrier for other compounds present in the standard solution and protecting the peptide. After 4 h, with 45% of proteins, this activity was almost 2 log_10_ higher than that of the initial solution. Once the nisin solution was not pure, it is possible to suggest that the BC may have acted as a filter. This barrier enhanced the nisin activity and, as a consequence of the nisin loading, a stable N–BC system formed. The N–BC could create meaningful material for pharmaceutical and food applications.

## 1. Introduction

Antimicrobial peptides are synthesized by several microorganisms. Nisin is a lantibiotic peptide, with 34 amino acids, and is secreted by Lactococcus [1,2,3,4]. The antimicrobial property of nisin is attributed to pore formation in the cell membrane of microorganisms, with specific binding to the lipidic precursor of the cell wall, attached to the membrane [4,5].

Nisin is considered safe by the World Health Organization (WHO) and the Food and Drug Administration (FDA-United States), being used initially as a food additive [6,7]. Further, its antimicrobial action encourages the clinical use of nisin, whether in topical or systemic therapies, due to its broad-spectrum activity and lower probability of developing microbial resistance [6,7,8]. The application of nisin extends to several medical areas, from mastitis to oral and gastrointestinal diseases [5,9,10].

Bacterial cellulose (BC) is a polysaccharide that is extracellularly secreted by several microorganisms, such as *Agrobacterium, Rhizobium*, *Escherichia*, *Sarcina*, and *Acetobacter* [11,12,13]. Namely, *Komagataeibacter xylinus* is a non-pathogenic Gram-negative bacterium that can produce significant amounts of cellulose [14]. BC is a linear glucose polymer, formed by a matrix of nanofibers, giving it porous characteristics in a three-dimensional network structure. Although its structure resembles vegetable cellulose, bacterial cellulose presents a high degree of purity, crystallinity, tensile strength, and high-water absorption [15].

Due to its biocompatibility and non-toxicity, the BC application has been directed to medical devices and tissue engineering [16]. Additionally, owing to its structural model, BC has been proposed as an ideal dermal substitute that is capable of inducing the direction of cells for repair in tissue reconstruction [17,18]. BC has been used as wound dressing because it forms a physical barrier against infections. It also allows gas exchange, absorbs exudates, keeps the wound moist to favor tissue reconstitution and it can be easily removed [19].

The incorporation of biomolecules into BC has been studied both by increasing its antibacterial or enzymatic properties and by providing a control release system [20,21]. For instance, nisin immobilization in solid matrices such as bacterial cellulose membranes could control its release [22]. Due to these properties, our research group previously evaluated the antimicrobial and antioxidant activity of nisin loaded into BC [23]. Since the work yielded satisfactory results, our group has provided a supplementary study. For this reason, this study has evaluated BC membranes as carriers for nisin regarding the morphological characteristics, cytotoxicity, and stability of the developed system.

## 2. Materials and Methods

### 2.1. Materials

The standard nisin and the bicinchoninic acid kit were purchased from Sigma-Aldrich (São Paulo, Brazil). All other reagents were of analytical grade.

### 2.2. BC Production and Purification

BC was produced by *Komagataeibacter xylinus* ATCC 53582, using 24-well plates with Hestrin and Schramm medium. Each well was filled with 1 mL and incubated at 30 °C in static conditions for seven days. After that, membranes were immersed in a 2% sulfate dodecyl sodium (SDS) solution under stirring overnight and washed in running water. The bleaching process was carried out with 4% NaOH for one hour until reaching 60 °C. The BC membranes were washed in order to remove NaOH and sterilized at 121 °C for 15 min [23,24].

### 2.3. Total Proteins Quantification

Protein concentration was determined by a bicinchoninic acid assay [25]. Bovine serum albumin (BSA) with different concentrations from 0.1 to 1.0 mg/mL was used as the standard protein. Absorbance reading was performed on 96-well microplates with a wavelength of 562 nm, by spectroscopy (Infinite M200 PRO, RCHISTO, Barueri, Brazil). Analyses were performed in triplicate, and the mean of these absorbance values was used to determine protein concentrations.

### 2.4. Nisin Standard Curve by Agar Diffusion Assay

The standard nisin (Sigma-Aldrich, St. Louis, MO, USA—containing 2.5% nisin, with 1,000,000 IU/g in its composition) solution was prepared by dissolving 1 g of nisin in 10 mL of phosphate buffer solution (PBS-pH 7.0), as in Table 1. The solution was centrifuged at 13,201 g for 10 min at 10 °C and the supernatant was collected and filtered in a 0.22 µm membrane (Millipore, Burlington, MA, USA). The nisin standard curve was evaluated by an agar diffusion assay. The nisin bioindicator *Lactobacillus sakei* ATCC 15521 was used for the agar diffusion assay [6,23].

The concentrations of standard nisin were related by the diameter of the inhibition halo (H, mm), and the activity of nisin was determined and expressed in arbitrary units per mL (AU/mL). The activity of nisin was based on the dilution of the standard nisin calibration curves. The correlation between AU/mL and international units per mL (IU/mL) was 1.09 ± 0.17 AU to 1.0 IU (40 IU = 1 µg of pure nisin A) [14,26].

### 2.5. Evaluation of Nisin Loaded into BC by Time

A dose of 250 µg/mL of nisin was employed based on previous studies from our research group [23]. The BC membranes were arranged in 24-well plates and 1.0 mL of nisin solution (250 µg/mL) was added in each well. The plate was placed on a shaker (NT 715, Nova Tecnica) at 25 °C, under 100 rpm for 4, 8, 12, 18 and 24 h. After each period, residual samples were collected in order to determine the nisin load efficiency by the concentration of proteins and nisin activity. The BC membranes were collected and evaluated by the agar diffusion assay for the measurement of the nisin activity.

The residual samples were evaluated by the agar diffusion assay and the total protein method. The load efficiency (LE) of nisin was evaluated by the total protein method and calculated by the following Equation (1), which was elaborated by the authors:

(1) $$\%\mathrm{LE}=\frac{\mathrm{Protein}\;\mathrm{in}\;\mathrm{the}\;\mathrm{initial}\;\mathrm{solution}-\mathrm{Protein}\;\mathrm{in}\;\mathrm{the}\;\mathrm{Residual}\;\mathrm{Solution}\;}{\begin{array}{c} \mathrm{Protein}\;\mathrm{in}\;\mathrm{the}\;\mathrm{initial}\;\mathrm{solution} \end{array}}\times 100$$

### 2.6. Stability Test by Agar Difusion Assay

In this test, *Staphylococcus aureus* ATCC 10390 and *Lactobacillus sakei* ATCC 15521 were utilized. *S. aureus* was grown in Tryptic Soy Broth (TSB) supplied by Becton, Dickinson and Company (BD Difco^TM^, Franklin Lakes, NJ, USA) for 24 h at 37 °C, whereas *L. sakei* was cultivated in De Man, Rogosa and Sharpe broth (MRS) supplied by Sigma-Aldrich Corporation (Sigma-Aldrich, St. Louis, MO, USA) for 24 h at 30 °C. All the media were prepared in distilled water and autoclaved at 121 °C for 30 min.

After the growth, the microorganisms were counted by the Pour Plate technique and the sample with 10^6^ UFC/mL was selected to carry out the agar diffusion assay. For *S. aureus*, an amount of 20 mL of TSB agar was placed in a Petri plate. After the solidification, 1 mL was placed on the agar surface. For *L. sakei*, an amount of 20 mL of MRS agar was placed in a Petri plate. After the solidification, 1 mL (10^6^ UFC/mL) was also placed on the agar surface.

The BC membranes loaded with nisin (N–BC) were kept in Petri plates and incubated at different temperatures (4, 25, 37 °C)—pre-set temperature conditions for stability evaluation—for 100 days. The samples were collected each day and the nisin activity was evaluated by the agar diffusion assay, adapted from ISO 20645, with *L. sakei* and *S. aureus*.

The BC was placed on an agar surface and the possible clear zones were observed after its incubation at 37 °C for 24 h. The presence of a clear halo that formed around BC was measured and indicated the antimicrobial activity [1].

### 2.7. Cytotoxicity Assay

The standard nisin and N–BC at the best time and with different concentrations of 7, 15, 31, 62, 125, 250 µg/mL were arranged in 24-well plates with 500 µL of DMEM (Dulbecco’s modified eagle’s medium, Low Glucose, Sigma^®^), with the addition of 10% fetal bovine serum (FBS, Sigma^®^) and 1% antibiotic (PEN/STREP, Sigma^®^) in each well. In each well, there were 75,000 L929 fibroblast cells. The plates were incubated at 37 °C in an atmosphere of 5% CO2 for 24 and 48 h. The assay was performed in triplicate and for positive control, PBS and DMEM were used.

After 24 and 48 h, cell viability was determined by the mitochondrial activity of the cell culture by the colorimetric assay MTS [3-(4,5-dimethylthiazol-2-yl)-5-(3-carboxymethoxyphenyl)-2-(4-sulfophenyl)-2H-tetrazolium]. Absorbance readings were taken at 490 nm, after 1 h of incubation of the cells with the reagent [27,28]. The results are expressed as mean percentage ± standard deviation (SD) of viable cells in relation to the positive control.

### 2.8. Morphology

#### 2.8.1. Scanning Electron Microscopy (SEM)

The BC and N–BC (with 250 µ/mL of nisin) were stored at −80 °C for 24 h and lyophilized for 48 h. BC and N–BC were set with carbon tape and metallized for 2 min (DH-29010SCTR Smart Coater). SEM images were observed on the scanning electron microscopy equipment (JEOL, Tokyo, Japan, Model IT200) and obtained using an accelerating voltage of 20 kV.

#### 2.8.2. X-ray Microtomography (μCT)

This equipment is responsible for analyzing pore size, porosity (%), and the interconnectivity of the pores in the membranes [29]. The membrane pictures were captured by X-ray microtomography (Bruckermicro CT-SkyScan 1174, Kontich, Belgium). The X-ray source was 29 kV and 661 mA. Here, 3D virtual models, representative of various sections of membranes, were built, and the data were mathematically managed by CT Analyzer software, v. 1.13.5.2.2.8.

### 2.9. Statistical Analyses

Differences among nisin concentrations are presented as mean ± standard deviation. ANOVA (analysis of variance) was used for parametric data, followed by Duncan or Scheffe tests for multiple comparisons. A *p* value < 0.05 was accepted as statistically significant. The data were analyzed using Statistica^®^8.0 (Statsoft software, Tulsa, OK, USA).

## 3. Results and Discussion

### 3.1. Nisin Standard Curve

**Figure 1:**
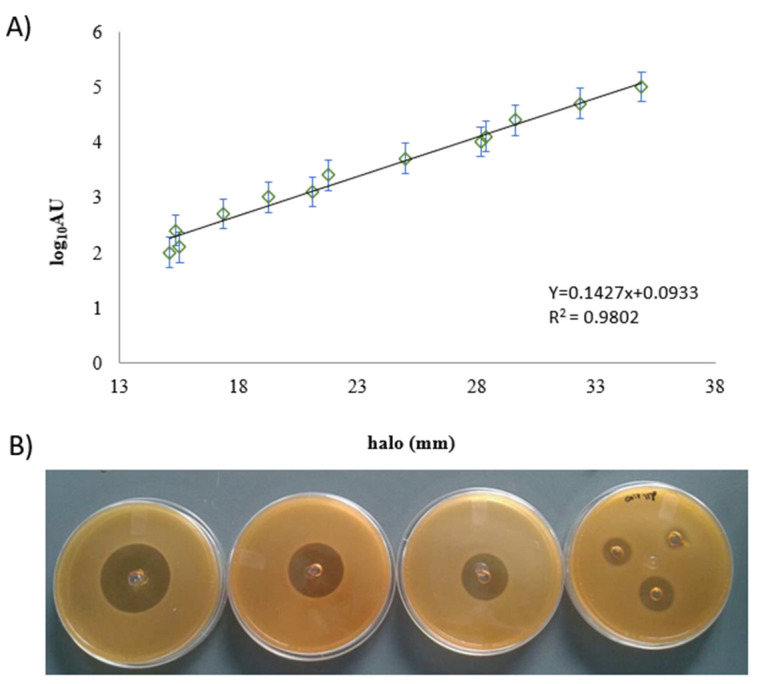
(**A**). Nisin standard curve by agar diffusion assay. (**B**) Agar diffusion assay image to represent the diameter of the inhibition halo.

For the nisin standard curve, zones of inhibition showed different sizes of halo ranging from 14 to 36.30 mm. Each halo corresponded to one concentration of nisin ranging from 2.5 µg/mL to 2500 µg/mL, as seen in Figure 1.

The standard curve demonstrates the sensibility of the bioindicator *L. sakei* even when it was exposed to low nisin concentration. This finding is important in establishing the relationship between the amount of nisin and the method that was used [30]. Nisin is a soluble peptide and its low molecular weight facilitates its diffusion in other matrices, supplementing the antimicrobial activity [31,32].

### 3.2. Evaluation of Nisin Loading in BC by Time

The nisin concentration was set at 250 µg/mL and we were able to observe the loading efficiency (%LE) by amount of protein (mg/mL) and nisin activity (log_10_AU) at different periods (Table 2). The protein concentration was not directly proportional to AU. Despite different incubation periods, there was little variation in nisin titers as observed in Table 2. These results are key to the definition of the best time for the load efficiency. After 4 h, the N–BC with 45% proteins was almost 2 log_10_ higher than the initial solution. Once the nisin solution was not pure, it is possible to suggest that the BC may have acted as a filter, separating the nisin from the other components and maybe causing overly strong adsorption kinetics in 4 h [33].

The same observation was reported by Ataide and colleagues [20]; they evaluated the enzyme bromelain loaded into the BC membranes, and its release. They noticed a threefold increase in the specific bromelain activity when compared to the initial solution, with 31% of proteins when bromelain was loaded into BC, showing the selective behavior of the BC membrane.

Jorge et al. [34] studied a system based on BC for the stabilization of insulin. The results indicate that BC was able to enhance permeation. Nguyen et al. [8] also evaluated the nisin loaded in the BC membranes. However, they reported 6 h as the best period for nisin incorporation in the BC membranes, two hours longer than in our study. Moreover, Nguyen and colleagues used 600 IU/mL in the initial solution, and we experimented with 10,000 AU/mL (4 log_10_AU).

According to Moniri et al. [35], BC has been extensively studied for controlled drug delivery as its structure is suitable for the dispensing of biomolecules.

This structure, which consists of nanofibers, as reported in the SEM section, probably explains the enhancement of nisin’s activity when loaded into BC. The BC nanofibers could act as a filter, retaining big molecules of dirt from the nisin solution and selectively allowing the absorption of the low-weight molecules, such as nisin.

### 3.3. Stability Test by Agar Diffusion Assay

**Figure 2:**
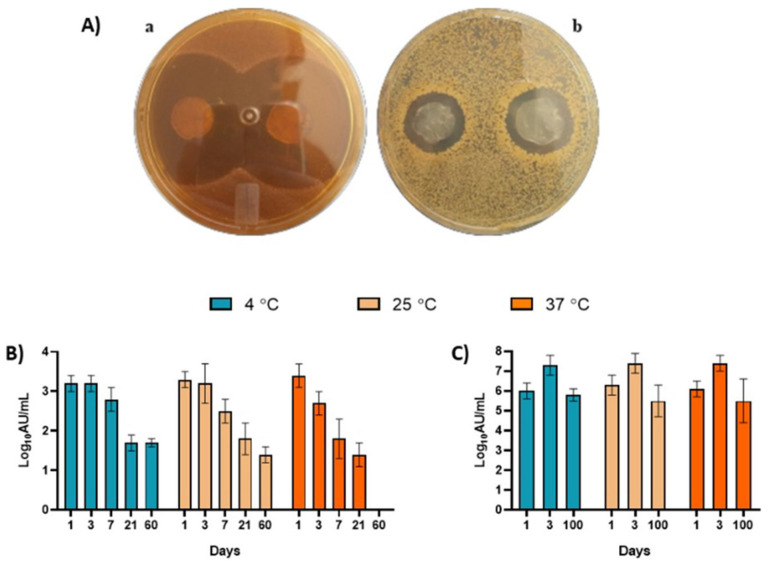
(**A**) Agar diffusion assay of the nisin loaded in BC (N–BC) against *L. sakei* (**a**) and *S. aureus* (**b**). (**B**) N–BC stability after storage at 4, 25 and 37 °C evaluated by agar diffusion assay against *S. aureus* ATCC 10390. (**C**) N–BC stability after storage at 4, 25 and 37 °C evaluated by agar diffusion assay against *L. sakei* ATCC 15521.

Figure 2A shows the qualitative agar diffusion test of N–BC against *L. sakei* (Figure 2(Aa)) and *S. aureus* (Figure 2(Ab)) after 7 days of storage at room temperature. Additionally, the quantitative tests of stability against *L. sakei* and *S. aureus* are shown in Figure 2B,C, respectively.

In general, the N–BC showed antimicrobial activity against *S. aureus* for 60 days at 4 and 25 °C. No antimicrobial activity was observed for 60 days at 37 °C. Despite having antimicrobial activity, N–BC was statistically stable for up to 7 days at 4 and 25 °C. At 37 °C, N–BC was stable for up to 3 days. After these periods of time, a significant decrease in activity was revealed.

On the other hand, N–BC was stable for up to 100 days against *L. sakei* at all incubation temperatures (Figure 2C), and no significant difference was seen.

By observing these results, we could hypothesize that if the N–BC system was used as wound dressing in tissues contaminated with *S. aureus*, it should be replaced—or even reapplied—after 7 days. Furthermore, if the N–BC system was used in food packaging, it should be considered for fresh food wrapping.

The process of nisin purification, described in the literature, shows that the activity of nisin can be reduced by fat clusters, salt concentrations and the aggregation of soluble proteins. It also shows that, after purification, nisin’s antimicrobial activity was increased [26].

Staphylococci are bacteria that live on the epithelial surfaces of humans and animals, being responsible for food poisoning and infections. With respect to bacterial resistance, nisin has been proven to be useful for limiting the development of antimicrobial resistance [36,37]. *S. aureus* also appears among the main bacteria responsible for secondary infections in burns [38,39].

Nisin’s antimicrobial activity against *S. aureus* generated a significant decrease in viable cells in a period of 1 day, and even inhibited biofilm formation [40]. Nisin encapsulation in nanofiber polymer provides stability to nisin for a period of up to seven days [39]. However, longer periods produced an inhibition zone inferior to what is presented in our study.

### 3.4. Cytotoxicity Assay

**Figure 3:**
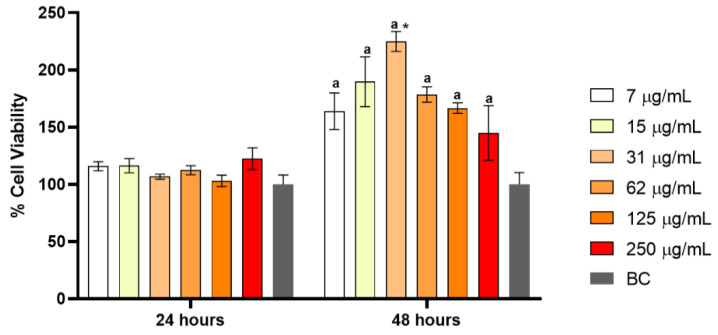
Cytotoxicity of nisin loaded in bacterial cellulose (N–BC) at concentrations of 7, 15, 31, 62, 125 and 250 µg/mL.; ^a^ *p* < 0.05 if compared to the respective concentrations at 24 h. * *p* < 0.05 if compared to the concentration of 7 µg/mL at 48 h.

Figure 3 shows the cytotoxicity data of N–BC at 24 and 48 h, at different concentrations. The fibroblast cells did not exhibit any significant change after 24 h of treatment. On the other hand, after 48 h, proliferation rates were 25% higher than those produced after 24 h, indicating the N–BC was able to stimulate cell proliferation, and no toxicity was reported.

Nisin is a safe molecule for both FDA and WHO [7,8,9,10,11,12,13,14,15,16,17,18,19,20,21,22,23,24,25,26,27,28,29,30,31,32,33,34,35,36,37], and BC is also considered a non-cytotoxic material [41,42]. Corroborating the Gao et al. [43] study, a cytotoxicity test of N–BC was performed in a co-culture and no cytotoxicity was observed. Proliferation in fibroblasts is important to accelerate the healing process.

The BC membranes loaded with nisin were used in large animals to treat surgical dehorn wounds [44]. The authors observed that the treatment with the BC membrane accelerated the healing process. Wound healing involves a dynamic set of tissue changes, important for maintaining the integrity of an organism. The authors also noted that BC may have created a barrier between the wound and the environment, preventing contamination.

In a previous work, dos Santos et al. [23] analyzed the antioxidant activity of nisin. The antioxidant activity may be related to the proliferation of fibroblasts since nisin has the potential to neutralize free radicals, facilitating cell proliferation.

### 3.5. Morphology

#### 3.5.1. Scanning Electron Microscopy (SEM)

**Figure 4:**
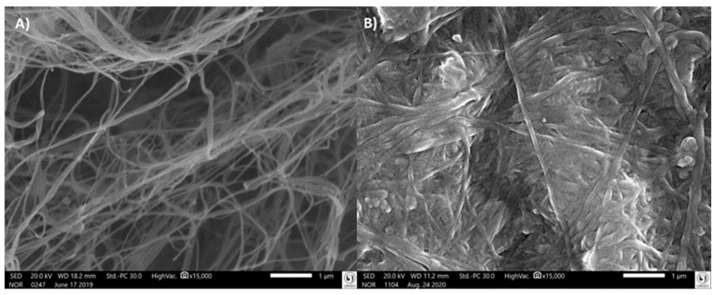
Scanning electron microscopy of the bacterial cellulose (BC) (**A**) and the bacterial cellulose loaded with nisin (N–BC) (**B**), at 15,000× magnification.

Figure 4A,B shows the BC without and with nisin, respectively. Comparing the morphology, it is possible to identify a similarity between the fibers on a micrometric and nanometric scale for both forms of BC. However, in N–BC, the fibers were more swollen than in BC, suggesting nisin incorporation.

The fiber network accounts for a large surface area and the porous structure of the BC facilitates the immobilization of nisin. In addition, this structure allows the diffusion of water, providing a functional stabilization of the biomolecules.

#### 3.5.2. X-ray Microtomography

The quantitative results of the structure were confirmed by X-ray microtomography assay, as presented in Table 3. The BC membranes presented more connectivity than the N–BC ones. Additionally, the presence of nisin decreased the porosity of membranes by 2%, supporting the SEM images. The pores were interconnected and distributed throughout the sample. The BC structure had a lower degree of anisotropy than the N–BC membrane.

Regarding membrane morphology, Gedarawatte et al. [45] performed the SEM test in nanocrystal BC containing nisin, and the presence of the antimicrobial was identified in the swelling of samples. The same behavior was observed in BC and nisin samples examined in co-culture by Gao et al. [43], reporting a large presence of nisin in the morphology of swelling fibers. The increase in fiber size indicated a high amount of antibacterial substance loaded in the BC membrane. Gao et al. [43] additionally observed that nisin can affect the BC fibers, resulting in a slight decrease in crystallinity and improving the antimicrobial property.

## 4. Conclusions

Here, we have proven the possibility of loading nisin into the BC membranes in 4 h, and also improving the antimicrobial activity with less protein, which suggests the BC membrane served as a selective barrier and it separated nisin from other components. This barrier enhanced nisin’s activity, and as a consequence of the nisin loading, a stable N–BC system was created and considered ideal for nisin delivery, with no toxicity and capacity to control the growth of microorganisms.

Thus, N–BC has the potential to be used as a dressing for skin wounds since nisin may prevent infections upon its release, requiring only a replacement at a point between 3 and 7 days.

Moreover, N–BC can be applied in in food packaging and other fields because its antimicrobial activity will probably reduce food deterioration and improve shelf-life.

## Figures and Tables

**Table 1:** Relation of concentration of standard nisin product, containing 2.5% of nisin, to its activity in AU and Log_10_AU.

Standard Nisin g/mL	0.1	0.01	0.001	0.0001
Standard Nisin Considering 2.5%	2500	250	25	2.5
Nisin Activity AU/mL	100,000	10,000	1000	100
Nisin Activity Log_10_ AU/mL	5	4	3	2

**Table 2:** The load efficiency (LE) of the nisin in bacterial cellulose by concentration of proteins compared to the nisin activity in different periods.

Time (h)	% LE	Log_10_AU
0	0	4.0 ± 0.00
4	43.7 ± 5.0	5.9 ± 0.12
8	47.6 ± 3.0	5.5 ± 0.35
12	42.9 ± 1.0	5.4 ± 0.08
18	40.0 ± 1.0	5.2 ± 0.12
24	17.8 * ± 3.0	5.5 ± 0.19

* *p* < 0.05 compared to each period (ANOVA test, followed by Duncan test).

**Table 3:** X-ray microtomography parameters of the bacterial cellulose (BC) and the bacterial cellulose with nisin (N–BC) membranes.

Parameter	BC	N–BC
Connectivity	22.421 ± 1.121	1.100 ± 0.55
Degree of anisotropy	0.692 ± 0.035	0.769 ± 0.038
Total porosity (%)	79.15 ± 3.96	77.95 ± 3.89
Open porosity (%)	79.17 ± 3.91	77.93 ± 3.89

Note: data were mathematically managed by CT Analyzer software, v. 1.13.5.2.2.8.

## Data Availability

The data presented in this study are available on request from the corresponding author.
